# Multiple bronchiolar adenomas with malignant transformation and CCNE1 mutation: a case report and literature review

**DOI:** 10.1186/s13019-021-01687-5

**Published:** 2021-10-18

**Authors:** Xiaojun Li, Yonghui Wu, Dayang Hui, Xiaoxuan Luo, Weibin Wu, Jian Zhang, Huiguo Chen

**Affiliations:** 1 Department of Cardiothoracic Surgery, The Third Affiliated Hospital, Sun Yat-Sen University, 600 Tianhe Road, Guangzhou, 510630 Guangdong People’s Republic of China; 2 Department of Pathology, The Third Affiliated Hospital, Sun Yat-Sen University, 600 Tianhe Road, Guangzhou, 510630 Guangdong People’s Republic of China; 3 Department of Radiation Oncology, The Third Affiliated Hospital, Sun Yat-Sen University, 600 Tianhe Road, Guangzhou, 510630 Guangdong People’s Republic of China

**Keywords:** Case report, Bronchiolar adenoma, Ciliated muconodular papillary tumor, Malignant transformation, CCNE1 mutation

## Abstract

**Background:**

Bronchiolar adenoma (BA) is a recently proposed diagnostic terminology, which is considered as the expansion of the concept of ciliated muconodular papillary tumors. BA is considered to be a benign neoplasm, but a few previous cases have been reported with the possibility of malignant transformation. Therefore, the genetic and histological nature of BA is controversial so far. We describe a rare case of multiple BAs with malignant transformation and CCNE1 (cyclin E1) mutation to increase the understanding of this disease.

**Case description:**

A 56-year-old woman was admitted to our hospital due to two ground-glass nodules (GGNs) in the left lung detected by chest CT without symptom. The pure GGN located in the upper lingual segment about 6 mm in diameter and another mixed GGN located in the dorsal segment about 7 mm. The two GGNs have been found a year ago without treatment, and the mixed GGN become larger to 8 mm with vacuole sign in the next year health checkup. We performed a wedge resection of the two nodules completely by video-assisted thoracoscopy (VATS). Postoperative pathology indicated that the pure GGN was atypical bronchial adenoma, while the mixed GGN was atypical bronchial adenoma with malignant transformation which was missed in frozen section. Gene mutations analysis by next-generation sequencing (NGS) showed CCNE1 gene mutation in both lesions, and her-2 mutation was identified in the mixed GGN. The programmed cell death 1 ligand 1 (PD-L1) expression analysis of tumor cells showed 0% and less than 1% in the pure GGN and the mixed GGN, respectively.

**Conclusion:**

BA is generally considered to be a benign tumor. The present study indicated that BA may be carcinogenic in atypical cases with some driver genes mutation and we should be vigilant for its potentiality of malignant transformation in clinical practice.

## Introduction

Bronchiolar adenoma (BA) is a recently proposed diagnostic terminology by Chang et al. [1], which is characterized as a putatively benign neoplasms corresponding to the anatomic epithelium of bronchioles. And the lesions currently designated as ciliated muconodular papillary tumor (CMPT) were proposed as a subset of BAs. According to the proposal by Chang et al. [1], the unified histological characteristic of BA is bilayered bronchiolar-type proliferation with a continuous layer of basal cells, which is considered to be the basis for BA identified as a benign tumor histologically. However, because of its rarity and potential malignant changes reported recently in few cases [2–5], the biological potential of BA remains controversial and unclear. Herein, we report a rare case of multiple BAs with malignant transformation and CCNE1 (cyclin E1) mutation, to increase the understanding of this disease and reduce missed diagnosis. To our knowledge, this is the first report on CCNE1 mutation and PD-L1 expression in BA.

## Case presentation

**Fig. 1:**
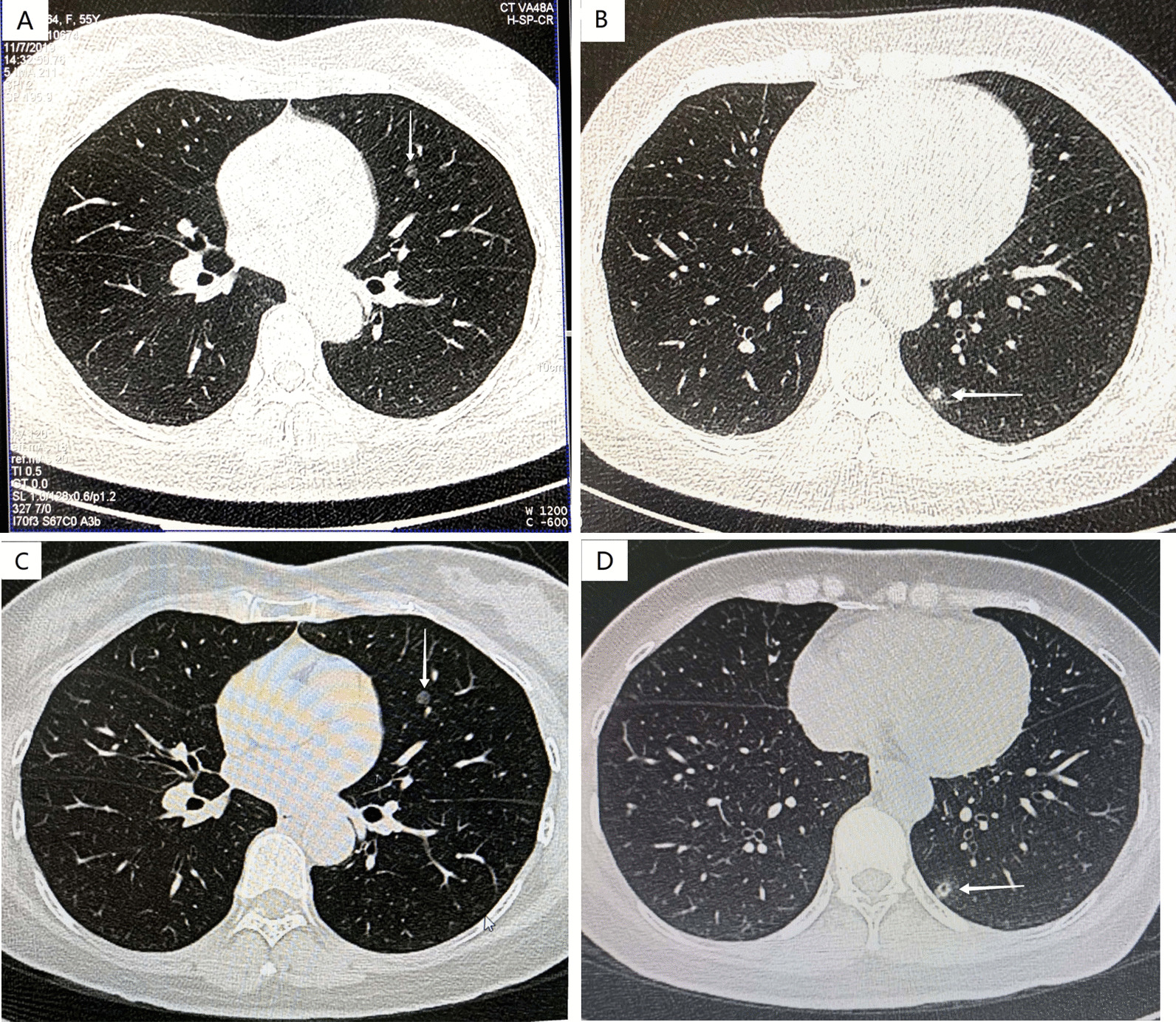
Computed tomography (CT) findings of BAs (arrows). **a** and **c**: a ~ 6 mm round pure GGN peripherally located in the lingual segment (**a**), and almost no change one year later (**c**). **b** and **d** a ~ 7 mm mixed GGN with irregular ill-defined peripheral opacity located in the posterior basal segment, adjacent to pleura (**b**), and slightly enlarged to 8 mm in diameter with vacuole sign one year later (**d**)

A 56-year-old woman was admitted to department of cardiothoracic surgery without any symptom in December 2020 due to two ground-glass nodules (GGNs) in the left lung detected by chest CT. There was no history of pneumonia, pleural effusion, thoracic surgery. A year ago, a chest computed tomography (CT) was performed in her annual checkup, and it showed two GGNs in the left lung. One pure GGN with round shape was peripherally located in the lingual segment about 6 mm in diameter (Fig. 1a), and the other mixed GGN with irregular ill-defined peripheral opacity and adjacent to pleura was located in the posterior basal segment about 7 mm in diameter(Fig. 1b). And then, the doctor advised her to observe. During the following year, the patient had no symptoms of respiratory discomfort. And the chest CT re-examination revealed that the pure GGN in the lingual segment did not change both in size or density (Fig. 1c), but the mixed GGN in the posterior basal segment were slightly enlarged to 8 mm in diameter with vacuole sign (Fig. 1d). No remarkable abnormality was found upon physical examination. Serum levels of tumor markers including squamous cell carcinoma antigen (SCC), carcinoembryonic antigen (CEA), cytokeratin 19-fragments (CYFRA21-1), neuron specific enolase (NSE) and cancer antigens 19-9 (Ca19-9) were within normal limits. Serum levels of infection markers including cryptococcal capsular polysaccharide antigen (CRAG), T-SPOT.TB, (1,3)-beta-D-glucan (BDG), C-react protein (CRP) and procalcitonin were all in the normal range. Because the mixed GGN was strongly suspected of lung cancer, the patient underwent video-assisted thoracoscopic wedge resection of the two nodules completely. Macrography, the pure GGN appeared as well-circumscribed tan-white to gray nodule, and the mixed GGN presented as faint yellow nodule with fuzzy boundary and a mucoid appearance. Frozen pathology of the mixed GGN showed abnormal hyperplasia of ciliated columnar epithelias with mild atypia, papillary or glandular architecture, and without nuclear division and hyperplasia of Clara cells. So, a diagnosis of adenoma was considered by frozen pathology. Because the pure GGN could be excluded invasive adenocarcinoma by CT and wedge resection can get sufficient incision margin, frozen pathology was not performed. The postoperative course was uneventful and the patient discharged hospital 3 days later.

**Fig. 2:**
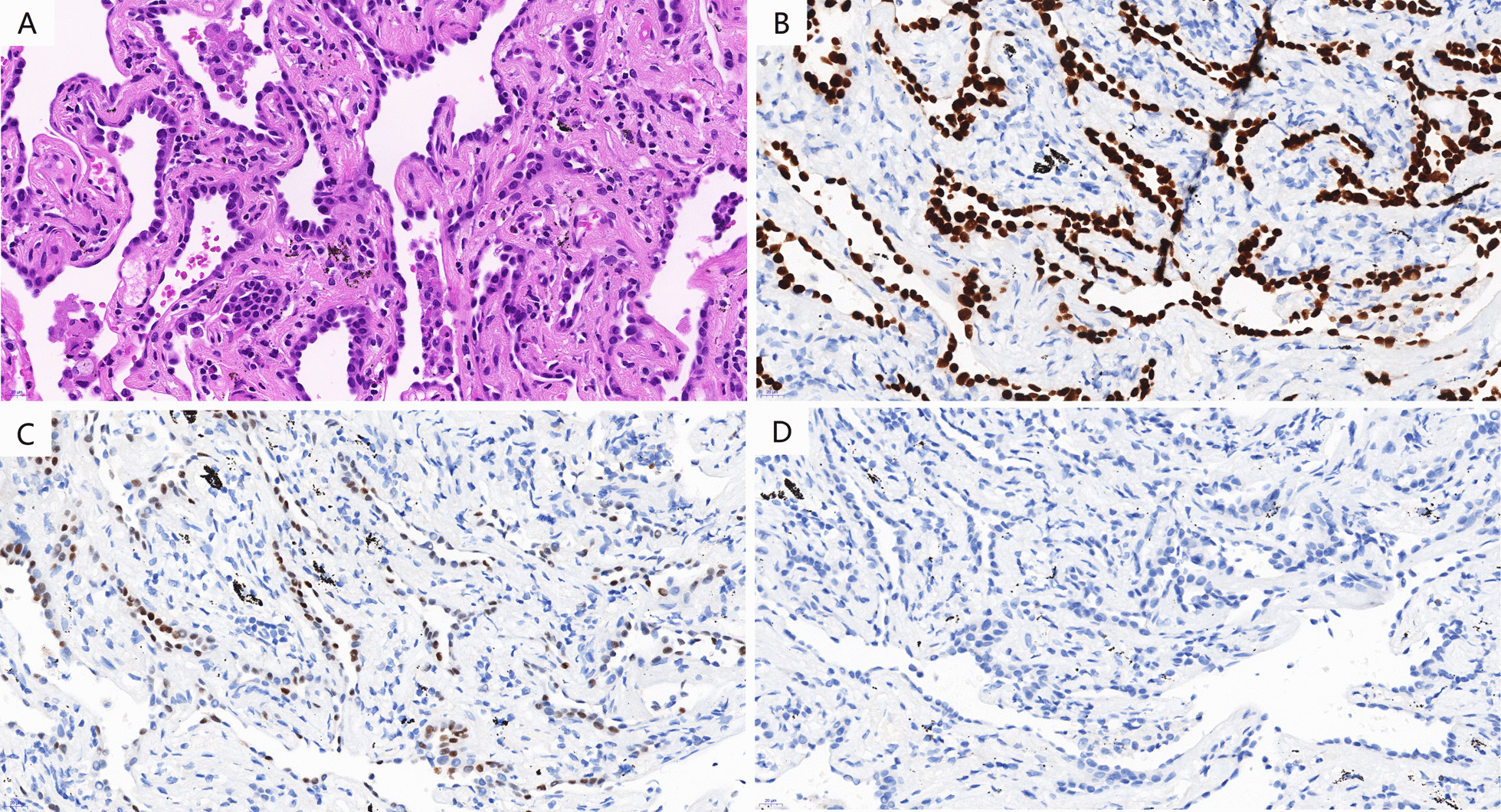
Pathological findings of pure GGN in the lingual segment of the left upper lobe. **a **Alveoli with single or double layers of columnar epithelial cells or flat cells (hematoxylin and eosin stain, magnification 400 ×). **b**, **c** and **d** immunohistochemical staining showed positive for TTF-1 and P63 (focal) and negative for CK5/6 (magnification 400 ×)

**Fig. 3:**
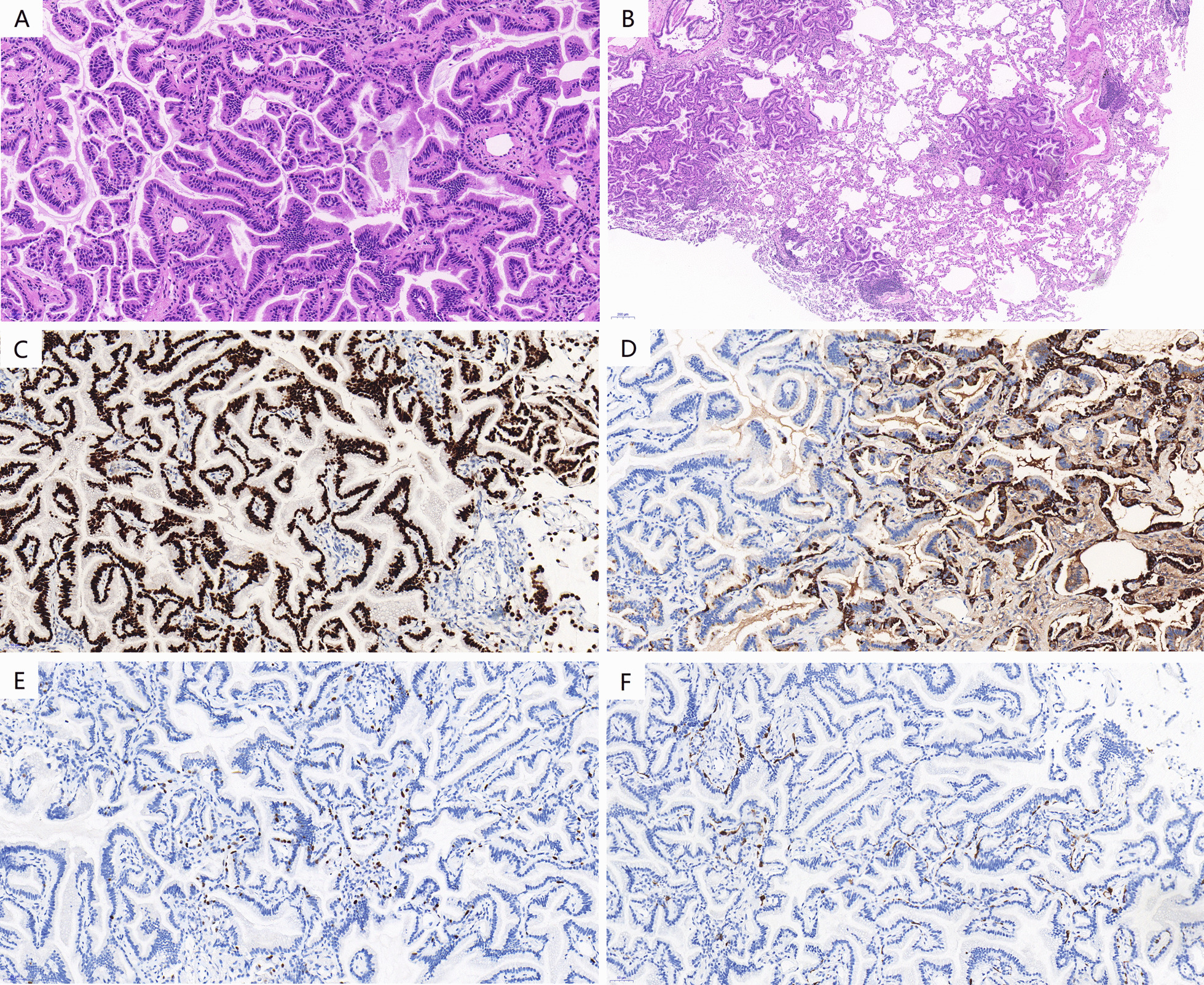
Pathological findings of mixed GGN in the posterior basal segment of the left lower lobe. **a** Predominantly papillary or glandular architecture covering single layer columnar epithelial cells with few ciliums (magnification 200 ×). **b** Multiple discontinuous skipping small lesions around the main lesion (magnification 50 ×). **c** Immunohistochemical staining showed positive for TTF-1 (magnification 200 ×). **d**–**f**: Immunohistochemical staining showed partial positive for Napsin-A, P63 and CK5/6 (magnification 200 ×)

In paraffin section, haematoxylin and eosin staining of the pure GGN showed there were alveoli of different sizes, with single or double layers of columnar epithelial cells or flat cells (Fig. 2a), of which the nuclei were small and the mitosis was rare. Immunohistochemical analysis showed that the tumor was positive for TTF-1 and P63 (focal) and negative for CK5/6 (Fig. 2b, c, d). Specific staining such as Periodic Acid-Schiff staining, silver hexosamine staining and acid-fast staining were negative. Final pathological diagnosis of the pure GGN was considered to be BA. And haematoxylin and eosin staining of the mixed GGN showed that there were predominantly papillary or glandular architecture covering single layer columnar epithelial cells with few ciliums (Fig. 3a), with disordered arrangement in local area, and the lesional cells showed slightly cytologic atypia but no increased mitotic activity. However, the boundary of tumor is not well defined, with multiple discontinuous skipping small lesions around the main lesion (Fig. 3b). Immunohistochemical analysis showed that the tumor was positive for TTF-1, Napsin-A (focal), P63 (partial) and CK5/6(a few) (Fig. 3c–f). The Ki-67 index was about 3%. So, final pathological diagnosis of the mixed GGN was considered to be BA with atypical hyperplasia and cancerization of adenocarcinoma in local area.

The two lesions underwent gene molecular analysis by next-generation sequencing (NGS) with a 71 gene panel (Hangzhou Repugene Technology Co., Ltd, Hangzhou, Zhengjian Province), and PD-L1 expression analysis by 22C3 pharmDx assay (Agilent Technologies, Carpinteria, CA). CCNE1 gene mutation (Exon7, c.476A > G) was identified in both lesions, with abundance of mutation for 48.83% in mixed GGN and 45.41% in pure GGN respectively. In addition, Her-2 mutation (Exon25, c.3089 T > G) was identified in mixed GGN with abundance of mutation for 1%. The PD-L1 expression of tumor cells shows 0% in the pure GGN and less than 1% in the mixed GGN.

## Discussion

The definition of bronchiolar adenoma was firstly proposed by Chang et al. [1] in 2018, and CMPT is considered to belong to a subset of BA. BA is composed of bilayered cellular proliferation with a continuous basal cell layer, which was highlighted by basal cell markers p40 and/or CK5/6 [1]. But in clinical practice, it was found that the tissue structure of some cases could not be completely conformed to the classical BA. Zhang et al. [6]. reported part of tumor only consist of a single layer of columnar or cubic epithelial cells in some cases of BA, without basal cell layer below, and this region is transitional to the tumor region with a double layer structure. Even some lesions were entirely composed of a single layer of columnar or cubic epithelial cells with glandular or papillary structures, which is smilar to the monolayer cell area of bronchial adenoma with monolayer cell changes. The authors defined such cases as atypical BA [6]. And in our case, the pure GGN showed there were alveoli of different sizes, with single or double layers of columnar epithelial cells or flat cells, and was positive for TTF-1 and P63 (focal). The mixed GGN showed that there were predominantly papillary or glandular architecture covering single layer columnar epithelial cells with a few ciliums without basal cell layer, and was positive for TTF-1, Napsin-A (focal), CK7, P63 (partial) and CK5/6(a few). So, we consider these two lesions as atypical BA.

Considering the spectrum of airway epithelium from the proximal bronchus to the peripheral alveolar structures, BA is considered to be a benign tumor [1], like a bronchial adenoma. However, it is still controversial whether BA has malignant potential. More and more cases with malignant transformation have been reported in the literature recently [3–5, 7]. Miyai et al. [3] reported a case of CMPT with malignant transformation of basal tumor cell components, and Chen et al. [7] descripted a case of mucinous adenocarcinoma caused by cancerization from CMPT and speculated CMPT is a precancerous lesion of mucinous adenocarcinoma. Han et al. [5] reported a case of BA losing continuity of the basal cell layer in the junctional zone between BA and IMA, and BA harbors the same KRAS mutation with the adjacent invasive mucinous adenocarcinoma. Therefore, a common histological feature of these cases is the absence of basal cell layer or the cancerization of basal cell, which indicated BA may be carcinogenic, especially in atypical cases. For the following reasons, we consider malignant transformation of the mixed GGN: First, the lesion lost the typical structures of the bronchioles and alveoli, with disordered morphology. Second, the boundary of the lesion is not clear, and there are discontinuous, skipping microscopic lesions. Third, the cells of the tumor had relatively swollen nuclei and were proliferating in a papillary or micropapillary manner.

Usually, it is extremely difficult to distinguish BA from AIS, minimally invasive adenocarcinoma (MIA) or adenocarcinoma in frozen section, especially adenocarcinoma [1]. Seven case of BA were misdiagnosed as adenocarcinomas in frozen sections. The reasons are as follows: first, some pathologists have insufficient understanding of the disease; second, some morphologic characteristics of BA are difficult to recognize in frozen sections; third, immunohistochemistry is required for differential diagnosis in some atypical cases. In this case, due to another case of typical BA has been identified 2 weeks ago in paraffin section, so the mixed GGN was diagnosed as BA quickly in frozen section. However, there are some findings to support malignant transformation as above. This significantly increases the difficulty of differential diagnosis in frozen section.

Some mutations of common driver genes for lung adenocarcinoma also have been identified in BAs, such as EGFR, KRAS and BRAF [1, 8, 9]. Different from the high frequency of EGFR mutation in lung cancer, a high percentage (50%) of BRAF mutation was found in BA [8]. Meanwhile, KRAS mutation has been found in 24% of BA [1]. In this case, only CCNE1 gene mutation was identified in both lesions and her-2 mutation was identified in mixed GGN. The protein encoded by CCNE1 gene participates and plays an important role in G1/S cell-cycle regulation. Recent study has found that the CCNE1 gene could serve as an oncogene and a poor prognostic factor in ovarian cancer and lung cancer [10–12]. However, CCNE1 gene mutation has not been reported in BA or CMPT so far. Further studies are needed to confirm whether CCNE1 promotes BA canceration.

## Conclusion

In summary, we report an extremely rare case of multiple BAs with malignant transformation and CCNE1 mutation, therefore we speculate BA may be carcinogenic in atypical cases with some driver genes mutation and should be vigilant in clinical practice. More data accumulation is needed to confirm the genetic, histological nature and prognosis of BA.

## Data Availability

All data in the study are included in this published article.

## Abbreviations

BA: Bronchiolar adenomas
CMPT: Ciliated muconodular papillary tumors
CCNE1: Cyclin E1
GGNs: Ground-glass nodules
PD-L1: Programmed death-ligand 1
